# A low initial serum sodium level is associated with an increased risk of overcorrection in patients with chronic profound hyponatremia: a retrospective cohort analysis

**DOI:** 10.1186/s12882-017-0732-1

**Published:** 2017-10-18

**Authors:** Sae Aratani, Masahiko Hara, Masahiko Nagahama, Fumika Taki, Miyuki Futatsuyama, Shuichi Tsuruoka, Yasuhiro Komatsu

**Affiliations:** 1 Department of Nephrology, St. Luke’s International Hospital, Tokyo, Japan; 2 Department of Nephrology, Graduate School of Medicine, Nippon Medical School, 1-1-5 Sendagi, Bunkyo-ku, Tokyo, 113-8603 Japan; 3 Department of Cardiovascular Medicine, Osaka City University Graduate School of Medicine, Osaka, Japan

**Keywords:** Chronic profound hyponatremia, Overcorrection, Risk factor

## Abstract

**Background:**

Even with abundant evidence for osmotic demyelination in patients with hyponatremia, the risk factors for overcorrection have not been fully investigated. Therefore the purpose of this study is to clarify the risks for overcorrection during the treatment of chronic profound hyponatremia.

**Methods:**

This is a single-center retrospective observational study. We enrolled 56 adult patients with a serum sodium (SNa) concentration of ≤125 mEq/L who were treated in an intensive care unit by nephrologists using a locally developed, fixed treatment algorithm between February 2012 and April 2014. The impact of patient parameters on the incidence of overcorrection was estimated using univariable and multivariable logistic regression models. Overcorrection was defined as an increase of SNa by >10 mEq/L and >18 mEq/L during the first 24 and 48 h, respectively.

**Results:**

The median age was 78 years, 48.2% were male, and 94.6% of the patients presented with symptoms associated with hyponatremia. The initial median SNa was 115 mEq/L (quartile, 111–119 mEq/L). A total of 11 (19.6%) patients met the criteria for overcorrection with 9 (16.0%) occurring at 24 h, 6 (10.7%) at 48 h, and 4 (7.1%) at both 24 and 48 h. However, none of these patients developed osmotic demyelination. Primary polydipsia, initial SNa, and early urine output were the significant risk factors for overcorrection on univariable analysis. Multivariable analysis revealed that the initial SNa had a statistically significant impact on the incidence of overcorrection with an adjusted odds ratio of 0.84 (95% confidence interval, 0.70–0.98; *p* = 0.037) for every 1 mEq/L increase. Additionaly, the increase in SNa during the first 4 h and early urine output were significantly higher in patients with overcorrection than in those without (*p* = 0.001 and 0.005, respectively).

**Conclusions:**

An initial low level of SNa was associated with an increased risk of overcorrection in patients with profound hyponatremia. In this regard, the rapid increase in SNa during the first 4 h may play an important role.

## Background

Hyponatremia, defined as a serum sodium (SNa) concentration of <135 mEq/L, is a water balance disorder, with a relative excess of body water in relation to sodium [1]. Some reports have classified SNa ≤ 125 mEq/L as “profound” hyponatremia [2, 3]. Since profound hyponatremia is also associated with fatal complications such as brain herniation, appropriate treatments for hyponatremia have been discussed intensively worldwide [4–6]. For example, urgent therapy using hypertonic saline is justified in life-threatening conditions caused by hyponatremia complicated with seizures and coma [2, 7, 8]. In contrast, the problem of overcorrection makes the treatment of hyponatremia difficult because it can provoke osmotic demyelination (ODS), which is a dreaded neurologic complication associated with high mortality [9]. Therefore, the international guidelines recommend limiting the increase of SNa to within 10 mEq/L and 18 mEq/L during the first 24 and 48 h, respectively, from the initiation of treatment for hyponatremia [2, 7]. To achieve the ideal correction rate of hyponatremia, several strategies including administration of desmopressin acetate (DDAVP), 3% hypertonic saline in combination with DDAVP, and re-lowering the SNa with 5% dextrose in water (D5W) have been introduced [10–13]. However, there is a paucity of literature on the risk factors for overcorrection [14], especially in “Chronic” profound hyponatremia, which carries a greater risk of neurologic complications from overcorrection, even though several risk factors for ODS have been identified, including SNa ≤ 105 mEq/L at presentation, overcorrection itself, malnutrition, hypokalemia, and liver disease [15–22]. We hypothesized that the identification of risk factors for overcorrection can improve the safety and efficacy of the treatment of chronic hyponatremia.

## Methods

### Aims

The aim of this study was to determine the risks for overcorrection during the treatment of chronic profound hyponatremia to provide physicians with additional information on the safe and ideal treatment of hyponatremia.

### Setting

This was a single-center retrospective cohort study, conducted by reviewing the medical records of a 520-bed teaching hospital (St. Luke’s International Hospital).

### Study patients

Consecutive patients with chronic profound hyponatremia, who were attended to by nephrologists, and treated in an intensive care unit (ICU) between February 2012 and April 2014, were included. Chronic hyponatremia was defined as hyponatremia that was documented to exist >48 h. If the duration could not be identified, we considered it as chronic [2]. Among the 64 patients who were admitted to the ICU with Sna ≤ 125 mEq/L, we excluded 8 who were not treated for hyponatremia by nephrologists. This exclusion ensured that treatment and data gathering were uniform throughout the study. Finally, 56 consecutive patients with SNa ≤ 125 mEq/L were included in the present study. The number of patients was confirmed using the treatment list of our department, as well as the electrical patient records of the hospital.

### Treatment protocol for Hyponatremia

**Fig. 1:**
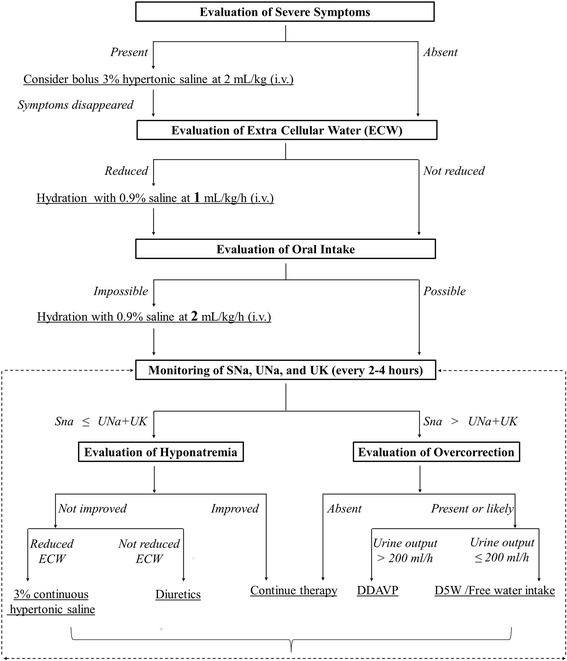
Standardized Therapeutic Protocol of Hyponatremia at Our Institution. Details of the protocol was explained in the methods section. All the patients were evaluated to identify the causes of hyponatremia and received cause-specific treatments. Bolus infusion of 3% hypertonic saline was administered for patients with severe symptoms, until the symptoms improved. DDAVP, desmopressin; D5W, 5% dextrose in water; ECW, extra cellular water; i.v., intravenous injection; p.o., per os; SNa, serum sodium; UK, urine potassium; and UNa, urine sodium

All patients were treated using our standardized therapeutic protocol for hyponatremia (Fig. 1). This therapeutic protocol fundamentally complied with the international treatment recommendations on chronic profound hyponatremia [2, 8], although it did involve an exceptionally aggressive strategy to avoid under-treatment. The steps in this protocol are carried out in sequence, rather than simultaneously. First, all the patients were evaluated to identify the causes of hyponatremia. This evaluation involved (1) an interview about the history of the present illness, (2) vital signs, (3) physical examination, (4) laboratory data, and (5) echocardiography, if appropriate. Urine osmolality ≦100 mOsm/kg suggested that the hyponatremia was caused by primary polydipsia or poor sodium intake. Patientsand received cause-specific treatments, such as water restriction for primary polydipsia, discontinuation of a drug that caused hyponatremia, and amelioration of physiological stimulation of antidiuretic hormone (ADH). In addition, if patients had severe symptoms, such as seizures, coma, or cardiorespiratory distress, an intravenous (i.v.) bolus infusion of 3% hypertonic saline of weight-based (2 mL/kg) amount was initiated until the symptoms improved. When the patients were relieved from these severe symptoms, they were assessed to determine the extracellular water (ECW) status. Patients were considered to have hypovolemic hyponatremia if the following findings were present, along with the discretion of the attending physician: history of gastrointestinal loss, reduced oral intake, thiazide usage, reduced turgor, or prolonged capillary refill time on physical examination, urine Na ≦ 30 mEq/L, and collapsed IVC on echography. Hydration with 0.9% saline at 1 mL/kg/h i.v. was initiated for patients who were diagnosed with reduced ECW. The administration speed of 0.9% saline was increased to 2 mL/kg/h when the patient could not take food orally.

Subsequently, we evaluated the SNa and urine sodium (UNa), urine potassium (UK), and urine output every 2–4 h during the first 48 h. Note that our remarkable degree of monitoring was to ensure safer and uniform treatment throughout the study.

We defined the initial 4-h urine output as “early urine output.” We compared the SNa to the total amount of UNa and UK (UNa + UK) to predict the possibility of a subsequent spontaneous increase in the SNa. In case the SNa was ≤ UNa + UK, hyponatremia was assumed not to be corrected spontaneously due to the absence of electrolyte-free water in the urine, and 3% hypertonic saline at a continuous rate of 0.5–1.0 mL/kg/h or diuretics were administered if hyponatremia persisted in the subsequent 4 h. Thus, 3% hypertonic saline was administered to patients with reduced ECW, and diuretics such as loop diuretics or tolvaptan were administered to patients without reduced ECW. Administration of 3% hypertonic saline was continued at 0.5–1.0 mL/kg/h until the SNa improved. We used this relatively aggressive protocol to avoid under-treatment, based on evidence that even mild hyponatremia is associated with increased morbidity and mortality in both hospitalized and ambulatory patients [23, 24].

On the contrary, if the SNa was > UNa + UK, SNa was expected to increase spontaneously by free water diuresis as the urine contained electrolyte free water. In such a situation, there was a subsequent possibility of overcorrection. We defined overcorrection as an increase of SNa by >10 mEq/L and >18 mEq/L during the first 24 and 48 h, respectively. Electrolyte-free water, D5W, or DDAVP was administered to prevent or manage the risk of overcorrection. DDAVP was administrated as an intranasal spray at a dose of 2.5–10 μg in patients with urine output >200 mL/h; urinary losses were replaced with D5W in patients with urine output ≤200 mL/h. If patients could consume free water orally, they were instructed to do so, instead of administering D5W infusion. We performed such interventions for patients who were likely to meet the criteria of overcorrection mentioned above as prophylactic interventions; those who met the criteria of overcorrection were administered these as therapeutic interventions.

### Statistical analysis

We set the primary endpoint of the present study as overcorrection of SNa, which was defined as an increase in the SNa by >10 mEq/L and >18 mEq/L during the first 24 and 48 h, respectively [2, 7]. Categorical data were represented as percentages (%) and continuous data as medians (25–75 percentiles). The impact of patient parameters on the incidence of overcorrection was estimated using univariable and multivariable logistic regression models, and represented as odds ratios (OR) with 95% confidence intervals (95% CI). In the multivariable model, the adjusted covariates included age, gender, presence of primary polydipsia, initial SNa, initial creatinine, and early urine output, and continuous infusion of 3% hypertonic saline; these constituted multivariable model 1. We selected these variables based on their clinical perspectives as only 11 primary events were identified in the present study. We also created a multivariable logistic regression model including 3 variables that demonstrated a *P* value of <0.05 in a univariable model; these constituted multivariable model 2. A P value <0.05 was considered statistically significant. For the comparison of patient parameters and treatment strategies, we divided our patients into 2 groups: the no-overcorrection group (*n* = 45) and overcorrection group (*n* = 11). The chi-square test or Wilcoxon rank sum test was used for comparisons of categorical and continuous data between the groups, respectively. We conducted all statistical analyses using the R software package (version 3.3.01, R Development Core Team, https://www.r-project.org/).

## Results

**Fig. 2:**
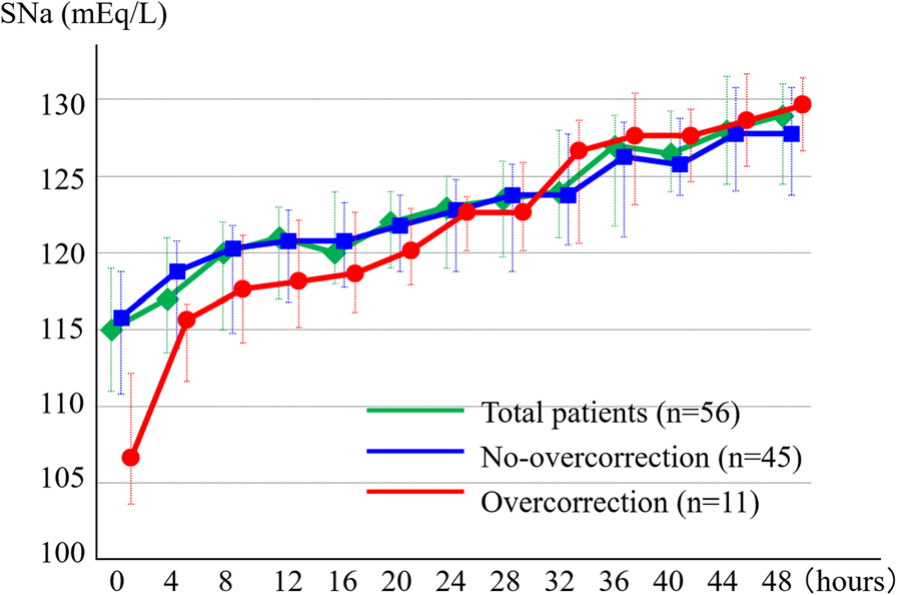
The result of SNa during the first 48 h. The results of SNa during the first 48 h are shown as medians (25–75 percentiles). The green line represents all patients, the blue line patients without overcorrection, and the red line patients with overcorrection. Error bars indicate the interquartile ranges. SNa, serum sodium

Table 1 shows the characteristics of the study population. The median age was 78 years; 48.2% were male. Most patients (94.6%) were symptomatic and 3 (5.4%) had severe symptoms. Among the 3 patients with severe symptoms, only one received a bolus infusion of 3% hypertonic saline, because the other 2 patients’ symptoms had resolved by the time the nephrologists evaluated them. In contrast, 16 patients received 3% hypertonic saline at a continuous rate (Table 3). There were multiple causes for hyponatremia; 36 (64.3%) patients were diagnosed to have hypovolemic hyponatremia and 21 (37.5%) with hyponatremia secondary to poor solute intake (Table 1). Table 2 shows the patients’ laboratory data at presentation.The initial median SNa was 115 mEq/L (quartile, 111–119 mEq/L). Table 3 shows the treatments and outcomes after the nephrology consultation. Hydration with 0.9% saline for volume repletion was performed in 36 (64.3%) patients, maintenance fluid infusion with 0.9% saline was performed in 32 (57.1%) patients, and 3% hypertonic saline was administered in 17 (30.4%) patients. Loop diuretics (i.v.) were administered in 4 (7.1%) patients and tolvaptan per os (p.o.) in 4 (7.1%) patients. Prophylactic and therapeutic interventions for overcorrection included the use of D5W in 18 (32.1%) patients, free water intake in 2 (3.6%) patients, and DDAVP in 11 (19.6%) patients. The mean initial time of DDAVP administration was 8 h (range, 7–12 h) after the presentation. A total of 11 (19.6%) patients met the criteria for overcorrection: 9 (16.0%) patients in 24 h, 6 (10.7%) in 48 h, and 4 (7.1%) in both 24 and 48 h. Fortunately, none of the patients developed ODS during or after hyponatremia treatment in this study. Figure 2 shows the time course of SNa levels during the first 48 h after the treatment.

**Table 1:** Patient characteristics

Parameters	Missing	Total (*n* = 56)	No-Overcorrection (*n* = 45)	Overcorrection (*n* = 11)	*p*-value
Age, years old	0	78 (60–83)	79 (63–84)	60 (48–82)	0.129
Male	0	27 (48.2)	22 (48.9)	5 (45.5)	0.838
Body weight, kg	0	52 (42–60)	50 (40–58)	56 (51–69)	0.049
Symptoms					
Severe symptoms					
Seizures	0	3 (5.4)	0 (0.0)	3 (27.3)	<0.001
Coma	0	0 (0.0)	0 (0.0)	0 (0.0)	–
Cardiorespiratory distress	0	0 (0.0)	0 (0.0)	0 (0.0)	–
Other symptoms					
Vomit	0	13 (23.2)	9 (20.0)	4 (36.4)	0.249
Nausea	0	9 (16.1)	7 (15.6)	2 (18.2)	0.832
Loss of appetite	0	10 (17.9)	10 (22.2)	0 (0.0)	0.085
Disorientation	0	34 (60.7)	28 (62.2)	6 (54.5)	0.640
Impairment of gait or fall	0	10 (17.9)	9 (20.0)	1 (9.1)	0.397
General fatigue	0	3 (5.4)	3 (6.7)	0 (0.0)	0.379
Asymptomatic	0	3 (5.4)	2 (4.4)	1 (9.1)	0.540
Cause of hyponatremia					
Primary polydipsia	0	6 (10.7)	2 (4.4)	4 (36.4)	0.002
Poor solute intake	0	21 (37.5)	17 (37.8)	4 (36.4)	0.931
Hypovolemic hyponatremia	0	36 (64.3)	30 (66.7)	6 (54.5)	0.452
SIADH	0	4 (7.1)	4 (8.9)	0 (0.0)	0.305
Physiological stimulation of ADH	0	11 (19.6)	9 (20.0)	2 (18.2)	0.892
CSW	0	2 (3.6)	2 (4.4)	0 (0.0)	0.476
Drug intoxication	0	10 (17.9)	8 (17.8)	2 (18.2)	0.975
Unidentified	0	1 (1.8)	1 (2.2)	0 (0.0)	0.618
Underlying disease					
Psychiatric disease	0	6 (10.7)	3 (6.7)	3 (27.3)	0.048
Schizophrenia	0	1 (1.8)	0 (0.0)	1 (9.1)	0.041
Anorexia nervosa	0	1 (1.8)	1 (2.2)	0 (0.0)	0.618
Depression	0	3 (5.4)	2 (4.4)	1 (9.1)	0.540
Development disorder	0	1 (1.8)	0 (0.0)	1 (9.1)	0.041
Infectious disease	0	14 (25.0)	13 (28.9)	1 (9.1)	0.174
Urinary tract infection	0	5 (8.9)	4 (8.9)	1(9.1)	0.983
Pneumonia	0	3 (5.4)	3 (6.7)	0 (0.0)	0.379
Thoracic empyema	0	1 (1.8)	1 (2.2)	0 (0.0)	0.618
Meningitis	0	2 (3.6)	2 (4.4)	0 (0.0)	0.476
Influenza	0	2 (3.6)	2 (4.4)	0 (0.0)	0.476
Cellulitis	0	2 (3.6)	2 (4.4)	0 (0.0)	0.476
HIV	0	1 (1.8)	1 (2.2)	0 (0.0)	0.618
Malignant disease	0	6 (10.7)	5 (11.1)	1 (9.1)	0.846
Stomach	0	1 (1.8)	1 (2.2)	0 (0.0)	0.618
Pancreatic	0	2 (3.6)	2 (4.4)	0 (0.0)	0.476
Renal	0	1 (1.8)	1 (2.2)	0 (0.0)	0.618
Prostatic		2 (3.6)	1 (2.2)	1 (9.1)	0.271
Alcohol abuse	0	4 (7.1)	3 (6.7)	1 (9.1)	0.780
Unidentified	0	28 (50.0)	23 (51.1)	5 (45.5)	0.737
Medication on admission					
Thiazide	0	7 (12.5)	5 (11.1)	2 (18.2)	0.525
Antipsychotic drug	0	2 (3.6)	2 (4.4)	0 (0.0)	0.476
DDAVP	0	2 (3.6)	2 (4.4)	0 (0.0)	0.476

*ADH* antidiuretic hormone, *CSW* cerebral salt wasting, *DDAVP* desmopressin, *HIV* human immunodeficiency virus, and *SIADH* syndrome of inappropriate antidiuretic hormone

Categorical variables are shown as numbers (percentages) and continuous variables as medians (25–75 percentiles)

**Table 2:** Laboratory data at presentation

Parameters	Missing	Total (*n* = 56)	No-Overcorrection (*n* = 45)	Overcorrection (*n* = 11)	*p*-value
Blood pressure, mmHg					
Systolic	0	130 (112–152)	127 (111–148)	142 (140–162)	0.034
Diastolic	0	70 (62–83)	70 (60–82)	78 (72–89)	0.137
Laboratory data					
Blood analysis					
Serum Na, mEq/L	0	115 (111–119)	116 (111–119)	107 (104–113)	0.012
Serum K, mEq/L	0	4.0 (3.4–4.5)	4.0 (3.7–4.7)	3.6 (3.3–4.1)	0.112
Serum BUN, mg/dL	0	12.5 (9.7–18.2)	14.2 (10.1–22.9)	10.1 (7.7–10.8)	0.023
Serum Cr, mg/dL	0	0.65 (0.44–0.94)	0.66 (0.46–1.12)	0.47 (0.42–0.73)	0.270
Serum TP, mg/dL	6	6.4 (6.0–7.3)	6.4 (6.0–7.3)	7.3 (6.3–7.8)	0.260
Serum Glu, mg/dL	8	123 (98–144)	124 (94–144)	120 (115–138)	0.741
Urinary analysis	0				
Urine Na, mEq/L	0	41 (20–65)	41 (24–66)	33 (17–46)	0.343
Urine K, mEq/L	0	28 (14–44)	33 (17–44)	13 (10–31)	0.058
Urine Na + K, mEq/L	0	74 (49–114)	75 (52–115)	57 (28–97)	0.101
Urine Osm, mOsm/kg H_2_O	1	332 (245–493)	336 (265–502)	217 (137–359)	0.029

*BUN* blood urea nitrogen, *Cr* creatinine, *Glu* glucose, *K* potassium, *Na* sodium, *Osm* osmolality, and *TP* total protein

Continuous variables as medians (25–75 percentiles)

**Table 3:** Treatments and outcomes

Parameters	Missing	Total (*n* = 56)	No-overcorrection (*n* = 45)	Overcorrection (*n* = 11)	*p*-value
Treatment of hyponatremia					
Hydration (0.9% saline)	0	36 (64.3)	30 (66.7)	6 (54.5)	0.452
Maintenance infusion (0.9% saline)	0	32 (57.1)	26 (57.8)	6 (54.5)	0.846
3% hypertonic saline					
Bolus infusion	0	1 (1.8)	0 (0)	1 (9.1)	0.041
Continuous infusion	0	16 (28.6)	15 (33.3)	1 (9.1)	0.111
Diuretics					
Loop diuretic (i.v.)	0	4 (7.1)	4 (8.9)	0 (0.0)	0.305
Tolvaptan (p.o.)	0	4 (7.1)	3 (6.7)	1 (9.1)	0.780
Intervention for overcorrection					
D5W	0	18 (32.1)	12 (26.7)	6 (54.5)	0.076
Instructed to free water intake	0	2 (3.6)	1 (2.2)	1 (9.1)	0.271
DDAVP	0	11 (19.6)	5 (11.1)	6 (54.5)	0.001
Time from admission, hours	0	8 (7–12)	9 (8–9)	8 (7–13)	0.853
Outcomes					
Osmotic demyelination	0	0 (0.0)	0 (0.0)	0 (0.0)	NA
Increase of serum Na, mEq/L					
At 4 h	5	2.0 (1.0–4.0)	2.0 (0.0–3.0)	6.5 (3.3–8.5)	0.001
At 24 h	0	8 (6–10)	7 (5–9)	12 (11–15)	<0.001
At 48 h	5	14 (12–17)	13 (11–15)	19 (17–23)	<0.001
Early urine output, mL/h	0	205 (114–386)	183 (85–303)	436 (196–998)	0.005
Urine output at 24 h, mL	0	2008 (1115–3101)	1640 (855–3005)	3725 (2229–4686)	0.001
Urine output at 48 h, mL	0	1930 (1416–3006)	1875 (1315–2655)	2695 (1723–3750)	0.073

*DDAVP* desmopressin, *D5W* 5% dextrose in water, *i.v.* intravenous injection *Na* sodium, and *p.o.* per os

Categorical variables are shown as numbers (percentages) and continuous variables as medians (25–75 percentiles)

Regarding the risk of overcorrection, Table 4 shows that primary polydipsia, initial SNa at presentation, and early urine output were significant risk factors on the univariable analysis. In addition, multivariable model 1 and 2 revealed that only the initial SNa at presentation had a statistically significant impact on the incidence of overcorrection with an adjusted OR of 0.84 (95% CI, 0.70–0.98; *p* = 0.037) for every 1 mEq/L increase in the multivariable model 1 after adjustment of all covariates listed in Table 3. Finally, we divided our study patients into the overcorrection and no-overcorrection groups, and compared the patient parameters, information on treatments and outcomes in Table 1, and the trend of SNa in Fig. 2 just for reference. It was noted that the increase in SNa during the first 4 h and early urine output were significantly higher for patients in the overcorrection group, as shown in Table 1 (*p* = 0.001 and 0.005, respectively).

**Table 4:** Impact of each variable on overcorrection

	Univariable		Multivariable model 1		Multivariable model 2	
Parameters	OR (95% CI)	*p*-value	adjusted OR (95% CI)	*p*-value	adjusted OR (95% CI)	*p*-value
Age	0.97 (0.93–1.01)	0.108	0.98 (0.92–1.05)	0.537	–	–
Male	0.87 (0.22–3.30)	0.838	0.63 (1.00–3.56)	0.603	–	–
Primary Polydipsia	12.3 (2.02–102.00)	0.009	2.41 (0.09–60.20)	0.576	3.25 (0.16–73.04)	0.432
Serum Na	0.83 (0.71–0.94)	0.006	0.84 (0.70–0.98)	0.036	0.86 (0.73–0.99)	0.041
Serum Cr	1.12 (0.51–2.04)	0.722	1.61 (0.72–4.01)	0.218	–	–
Early urine output^a^	1.41 (1.13–1.87)	0.006	1.30 (0.94–1.94)	0.149	1.26 (0.93–1.79)	0.163
3% hypertonic continuous infusion	0.20 (0.01–1.19)	0.142	0.54 (0.02–5.50)	0.629	–	–

*CI* confidence interval *Cr* creatinine, *Na* sodium, and *OR* odds ratio

Multivariable model 1 included all 7 variables shown in the Table. Multivariable model 2 included 3 variables that showed statistical significance on univariable analysis

^a^Odds ratio per 100 mL increase

In addition, among the 56 patients, 6 (11%) from the “no-overcorrection” group died during hospitalization due to infectious disease (*n* = 4) or cancer (*n* = 2). Both of these conditions may have caused hyponatremia in these patients. Among the 50 patients who were discharged alive, 12 were lost to follow-up partly because they were referred to their family doctors or because they cancelled their outpatient visits unexpectedly. Among the 38 patients who were ultimately followed-up at our institution, there were no re-hospitalizations due to hyponatremia within a median follow-up of 822 (quartile, 199–1411) days.

## Discussion

In this single-center retrospective observational study, we investigated the risk factors for overcorrection by enrolling 56 patients with chronic profound hyponatremia who underwent standardized therapeutic strategies including DDAVP administration at our institution. We found two clinically important observations. First, an initial low level of SNa was a statistically significant risk factor for overcorrection. Second, the rapid increase in the first 4 h of the treatment might be responsible for the overcorrection, as suggested by Fig. 2.

### Incidence of overcorrection and ODS

The incidence of overcorrection varies depending on its definition and mode of correction [2, 7]. A retrospective study that involved 412 patients with SNa < 120 mEq/L, in which overcorrection was defined as an increase in the SNa by >10 mEq/L during the first 24 h, reported that 114 (27.9%) patients were overcorrected [19]. The study, however, did not discuss the treatment strategies for hyponatremia in detail. Another retrospective study that included 62 patients concluded that 7 (11.3%) patients exceeded the increase of SNa by >12 mEq/L and 6 (9.7%) patients by 18 mEq/L during the first 24 and 48 h, respectively [14]. The treatment included administration of 3% hypertonic saline, D5W, and DDAVP, which were used in patients in whom overcorrection was likely or had occurred. The incidence of overcorrection in our study was consistent with that observed in these previous studies. We identified that 9 (16.0%) patients had an increase in the SNa by >10 mEq/L and 6 (10.7%) by >18 mEq/L during the first 24 and 48 h, respectively.

The international guidelines define overcorrection with a safety margin. We speculated that no ODS occurred mainly because the increase in SNa was relatively limited in the present study; even in patients with overcorrection, SNa increased by 12 mEq/L (quartile, 11–15 mEq/L) and 19 mEq/L (quartile, 17–23 mEq/L) during the first 24 and 48 h, respectively (Table 3). Similarly, in a previous retrospective study in which four of 37 patients (11%) with overcorrection developed ODS [25], the increases in SNa were 21 ± 5 mEq/L and 28 ± 8 mEq/L during the first 24 and 48 h, respectively. Therefore, we believe that minimizing SNa increases is essential to prevent ODS, even when overcorrection does occur.

### Risk of overcorrection

Risk factors for ODS included an initial SNa of <105 mEq/L and overcorrection. International guidelines recommend avoidance of overcorrection in the management of patients with hyponatremia; however, risk factors for overcorrection have not been fully investigated [2, 7]. In contrast, avoidance of overcorrection or ODS may lead to prolonged or inadequate management of patients with hyponatremia, leading to undercorrection in clinical practice. We need to develop an optimal treatment strategy to assure a safe correction rate of SNa to achieve an appropriate correction while avoiding overcorrection. With this point of view, we conducted this retrospective study to determine the risk factors for overcorrection and believe that our results will guide physicians in the management of these patients. Our study revealed that a low level of SNa at presentation was associated with overcorrection, with an adjusted OR of 1.19 for every 1 mEq/L decrease (0.84 for every 1 mEq/L increase). This meant that a 4 mEq/L lower SNa level doubled the risk of overcorrection. Although several previous retrospective studies have indicated that the SNa at presentation was lower in patients with overcorrection compared to those without, a direct association between the SNa levels and risk of overcorrection has not been evaluated previously [14]. Our results alert clinicians to be cautious of the incremental risk of overcorrection when patients present with low SNa levels.

### Mechanism and intervention of overcorrection

Regarding the mechanism of overcorrection, it is noteworthy that a rapid increase in the SNa levels during the first 4 h of treatment might be responsible for the overcorrection, as suggested in Fig. 2. In our study, patients with overcorrection showed a rapid increase in the SNa levels soon after hospitalization, with greater early urine output, compared to those without overcorrection. This could happen when factors stimulating ADH secretion subside following hospitalization [26]. For example, resolution of hypovolemia by administration of 0.9% saline, discontinuation of thiazides, and amelioration of pain or stress would easily suppress the release of ADH [27, 28], leading to free water diuresis. Thus, we speculated that the hospitalization itself strongly contributed to the suppression of ADH secretion and the resultant rapid increase in SNa, especially when the SNa levels were very low. Hence, it is clinically important to manage the risk of overcorrection during the early period following presentation to avoid a rapid increase in the SNa levels; this should be considered even if a reactive approach, such as the one described in our standardized therapeutic protocol (Fig. 1), is followed. Sood L et al. recently proposed a unique approach to control the correction rate of hyponatremia by administering DDAVP as the initial treatment along with 3% saline. Their study enrolled 25 patients and showed that the approach was an effective and safe strategy to manage water diuresis [10]. In that study, the mean increase in SNa was 5.8 ± 2.8 mEq/L and 4.5 ± 2.2 mEq/L during the first and second 24 h, respectively. No patient experienced overcorrection. On the contrary, in our study, 54.5% of patients in the overcorrection group received DDAVP administration and the mean time from admission to DDAVP administration was 8 h (range, 7–13 h) (Table 2). Our results indicate that early detection and cautious monitoring of the rapid increase in SNa during the first 4 h of treatment are important in terms of clinical implications, although there is no consensus on the optimum monitoring frequency. For example, hourly monitoring or early administration of DDAVP during the first 4 h could be a reasonable and effective strategy to prevent overcorrection in high risk patients with an initial SNa of <110 mEq/L.

### Study limitations

This study had some limitations. First, this was a single-center retrospective study with a small sample size of 56 patients. Some patients with chronic profound hyponatremia may have been admitted to the general ward and treated by general physicians, without the involvement of nephrologists. Second, the definition of overcorrection varies among studies [29]. Finally, we ourselves designed the algorithm used in hyponatremia treatment. The protocol basically complied with international guideline recommendations for the treatment of chronic profound hyponatremia, although we did employ an exceptionally aggressive strategy involving continuous administration of 3% hypertonic saline in some situations to avoid under-treatment [2, 8, 23, 24]. In addition, the purpose of this standardized algorithm was not to recommend our treatment protocol to other institutions, but to make our results comparable to other studies. The appropriateness of applying diagnostic algorithms to treat hyponatremia remains to be studied, and physiology-based treatment must always be kept in mind [30, 31]. Our data should be interpreted in the light of these limitations.

## Conclusions

The present study identified that an initial low level of SNa was associated with an increased risk of overcorrection in patients with profound hyponatremia. The rapid increase in SNa during the first 4 h may play an important role in overcorrection. It is also important to note that no ODS occurred in the present study, and that minimizing SNa increases is important to prevent ODS, even when overcorrection does occur.

## Abbreviations

ADH: Antidiuretic hormone
BUN: Blood urea nitrogen
CI: Confidence intervals
Cr: Creatinine
CSW: Cerebral salt wasting
D5W: 5% dextrose in water
DDAVP: Desmopressin
ECW: Extracellular water
Glu: Glucose
HIV: Human immunodeficiency virus
i.v.: Intravenous injection
ICU: Intensive care unit
K: Potassium
Na: Sodium
ODS: Osmotic demyelination
OR: Odds ratios
Osm: Osmolality
p.o.: Per os
SIADH: Syndrome of inappropriate antidiuretic hormone
TP: Total protein
UK: Urine potassium
UNa: Urine sodium
